# Correction: Dual bioresponsive antibiotic and quorum sensing inhibitor combination nanoparticles for treatment of *Pseudomonas aeruginosa* biofilms *in vitro* and *ex vivo*

**DOI:** 10.1039/d5bm90038g

**Published:** 2025-05-12

**Authors:** Nishant Singh, Manuel Romero, Alessandra Travanut, Patricia F. Monteiro, Elena Jordana-Lluch, Kim R. Hardie, Paul Williams, Morgan R. Alexander, Cameron Alexander

**Affiliations:** a School of Pharmacy, University of Nottingham University Park Nottingham NG7 2RD UK cameron.alexander@nottingham.ac.uk; b National Biofilms Innovation Centre (NBIC), School of Life Sciences, University of Nottingham, Centre for Biomolecular Sciences University Park NG7 2RD UK; c School of Life Sciences, University of Nottingham, Centre for Biomolecular Sciences University Park NG7 2RD UK

## Abstract

Correction for ‘Dual bioresponsive antibiotic and quorum sensing inhibitor combination nanoparticles for treatment of *Pseudomonas aeruginosa* biofilms *in vitro* and *ex vivo*’ by Nishant Singh *et al.*, *Biomater. Sci.*, 2019, **7**, 4099–4111, https://doi.org/10.1039/C9BM00773C.

The authors regret an inadvertent mistake was made during assembly of Fig. 4. This was due to selection of the wrong image from the many files recorded from replicate samples, each with widefield *Z*-stack images, at the time. The error arose from the wrong image for “ALGQSICIP” 24 h panel being included in Fig. 4A. To make sure the image used to obtain the data displayed by blue bars in graph 4B was correct, they have performed a new quantification of the normalised fluorescence intensity ratios of all conditions. The quantification of the correct image indicates no substantial differences in the fluorescence profiles to those shown in Fig. 4B. Accordingly, they have revised the images in Fig. 4 and the quantification of the fluorescence data and the corrected Fig. 4 is shown here.

**Fig. 4:**
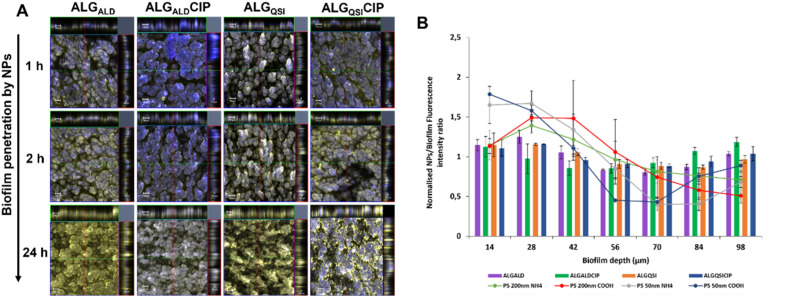
Penetration of *P. aeruginosa* biofilms by alginate nanoparticles. In (A), confocal laser scanning microscopy (CLSM) images show penetration of 6-amino fluorescein-ALG_ALD_, ALG_ALD_CIP, ALG_QSI_ and ALG_QSI_CIP NPs over time into PAO1-N biofilms stained with SYTO64 dye. The fluorescein labelled NPs are shown in yellow and the bacteria are stained blue. After incubating the biofilms for 1 h no penetration is observed as evident from the blue stained biofilm. The NPs begin to penetrate after 2 h of incubation as observed by the increase in yellow fluorescence from the biofilm structure. Within 24 h complete penetration to the biofilm bulk is observed, evident from the 3D *Z*-stack of the biofilm shown on the top and right of the main image. Scale bar: 100 μm. In (B), the penetration of the different ALG-based NPs within 24 h is quantified (bars), in contrast to the polystyrene (PS) NPs which are mostly present in the outer layer of the biofilm (28–42 μm depth, circles joined with lines). The fluorescence intensities of the ALG NPs and polystyrene NPs are normalised to the background fluorescence of the biofilm stain.

An independent expert has viewed the corrected images and raw data and has concluded that they are consistent with the discussions and conclusions presented.

The Royal Society of Chemistry apologises for these errors and any consequent inconvenience to authors and readers.

## Data availability

Data included in this manuscript (IR, NMR, DLS and image files) are available at https://rdmc.nottingham.ac.uk.

